# Influence of *TCF7L2* gene variants on the therapeutic response to the dipeptidylpeptidase-4 inhibitor linagliptin

**DOI:** 10.1007/s00125-014-3276-y

**Published:** 2014-06-07

**Authors:** Heike Zimdahl, Carina Ittrich, Ulrike Graefe-Mody, Bernhard O. Boehm, Michael Mark, Hans-Juergen Woerle, Klaus A. Dugi

**Affiliations:** 1 Drug Metabolism and Pharmacokinetics, Development, Boehringer Ingelheim Pharma GmbH & Co. KG, Birkendorfer Straße 65, 88397 Biberach/Riss, Germany; 2 Global Biometrics and Clinical Applications, Medicine, Boehringer Ingelheim Pharma GmbH & Co. KG, Biberach/Riss, Germany; 3 TA Metabolism, Medicine, Boehringer Ingelheim Pharma GmbH & Co. KG, Ingelheim, Germany; 4 Lee Kong Chian School of Medicine, Nanyang Technological University, Singapore, Republic of Singapore; 5 Diabetes, Endocrinology and Metabolism, Department of Medicine, Imperial College London, London, UK; 6 Department of Internal Medicine I, Ulm University Medical Centre, Ulm, Germany; 7 CardioMetabolic Diseases, Research, Boehringer Ingelheim Pharma GmbH & Co. KG, Biberach/Riss, Germany; 8 Corporate Division Medicine, Boehringer Ingelheim GmbH, Ingelheim, Germany

**Keywords:** Clinical science, DPP-4 inhibitor, Genetics of type 2 diabetes, Human, Linagliptin, Oral pharmacological agents, Pharmacogenomics, *TCF7L2*

## Abstract

**Aims/hypothesis:**

Individuals carrying variants of the transcription factor 7-like 2 gene (*TCF7L2*) are at increased risk for type 2 diabetes. These metabolic genetic risk factors have been linked to diminished pancreatic islet-cell responsiveness to incretins, thus pharmacological interventions aimed at amplifying endogenous incretin biology may be affected. However, clinical evidence from randomised controlled trials so far is lacking. We investigated the influence of *TCF7L2* risk alleles on the response to treatment with the dipeptidylpeptidase-4 (DPP-4) inhibitor linagliptin from four 24 week, phase III, placebo-controlled trials.

**Methods:**

Pharmacogenomic samples and clinical data were available from 961 patients with type 2 diabetes. Whole-blood DNA samples were genotyped for *TCF7L2* single-nucleotide polymorphisms in conjunction with assessments of 24 week changes in HbA_1c_.

**Results:**

Linagliptin lowered HbA_1c_ meaningfully in all three genotypes of rs7903146 (non-risk variant carriers CC [*n* = 356]: −0.82% [−9.0 mmol/mol], *p* < 0.0001; heterozygous CT [*n* = 264]: −0.77% [−8.4 mmol/mol], *p* < 0.0001; homozygous risk variant carriers TT [*n* = 73]: −0.57% [−6.2 mmol/mol], *p* < 0.0006). No significant treatment differences were seen between CC and CT patients, although HbA_1c_ response was reduced in TT compared with CC patients (~0.26% [~2.8 mmol/mol], *p =* 0.0182).

**Conclusions/interpretation:**

Linagliptin significantly improved hyperglycaemia in patients with type 2 diabetes both with and without the *TCF7L2* gene diabetes risk alleles. However, differences in treatment response were observed, indicating that diabetes susceptibility genes may be an important contributor to the inter-individual variability of treatment response.

**Electronic supplementary material:**

The online version of this article (doi:10.1007/s00125-014-3276-y) contains peer-reviewed but unedited supplementary material, which is available to authorised users.

## Introduction

Diabetes is a fast-growing global epidemic with an increasing prevalence worldwide [1]. Several genes have been associated with type 2 diabetes susceptibility or manifestation, including genes encoding receptors, transcription factors, cell cycle-associated proteins, modifiers of signal transduction, ion channels and others [2–4]. Recently, single-nucleotide polymorphisms (SNPs) of a gene encoding transcription factor 7-like 2 were shown to have the strongest known genetic risk factor for type 2 diabetes among all diabetes-associated gene SNPs [5, 6]. The risk of developing diabetes is twice as high in homozygous *TCF7L2* risk variant (rs7903146) carriers (TT) compared with non-risk carriers (CC) [7, 8]. The initial findings have been replicated in independent studies in multiple ethnic populations and were summarised in a large global meta-analysis [5]. Pharmacogenetic studies reported a significant association between *TCF7L2* risk variants and efficacy of sulfonylurea treatment, with a twofold greater likelihood of sulfonylurea treatment failure in *TCF7L2* risk carriers [9]. The mechanisms by which *TCF7L2* polymorphisms increase diabetes risk and affect the treatment response to insulin secretagogues were thought to be related to impaired incretin-induced insulin secretion, impaired suppression of glucagon or impaired glucagon-like peptide-1 secretion [10–13]. Depending on the underlying mechanism, the response to other insulin secretagogues, such as the novel class of dipeptidylpeptidase-4 (DPP-4) inhibitors, also may be affected.

Incretin hormones amplify the first phase of insulin secretion [14]. The advantage of incretin-based therapies, like orally active DPP-4 inhibitors, is that they have a glucose-dependent insulinotropic action with no intrinsic risk for causing hypoglycaemia. Linagliptin, a potent and selective inhibitor of DPP-4, improves glucose homeostasis in patients with diabetes by blocking the degradation of incretins and thus improving insulin secretion in a glucose-dependent manner [15, 16]. Linagliptin has been approved for the treatment of patients with type 2 diabetes [16, 17]. Since linagliptin and the high-risk polymorphisms of *TCF7L2* both affect the same process responsible for the first phase of insulin secretion, it can be hypothesised that the response to linagliptin therapy may differ in patients depending on their allele status. Therefore, we wanted to explore whether the efficacy response to linagliptin (i.e. change from baseline in HbA_1c_ or change from baseline in 2 h postprandial plasma glucose [PPG] after 24 weeks of treatment) is dependent on the *TCF7L2* genotype in a retrospective analysis of clinical data.

## Methods

### Data sources

**Fig. 1:**
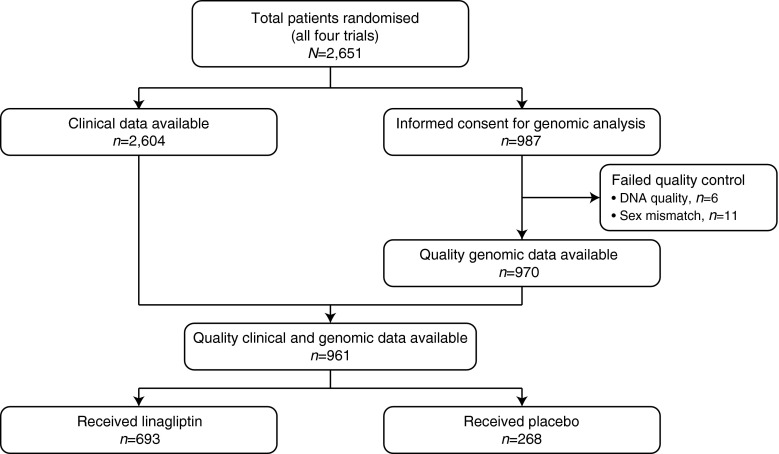
Sample selection for analyses of *TCF7L2* SNPs with clinical outcome

We conducted analyses of data from four phase III clinical trials—NCT00601250 [18], NCT00602472 [19], NCT00621140 [20] and NCT00641043 [21] (www.clinicaltrials.gov)—that evaluated the safety and efficacy of linagliptin, as monotherapy or in combination with other glucose-lowering therapy, in improving glycaemic control in patients with type 2 diabetes (Table 1). Patients were on stable doses of diabetes medications or, for NCT621140, on no medications except for linagliptin or placebo. Out of a total of 2,651 patients randomised to different arms in the four trials, 987 patients gave informed consent for pharmacogenomic analyses. Both clinical and pharmacogenomic data needed for the current analyses were available for 961 patients, of which 693 were treated with linagliptin and 268 received placebo (Fig. 1). Depending on the trial, patients continued to receive other glucose-lowering medication in accordance with the objectives of that trial. Demographic and baseline characteristics of the patients included in these analyses are given in Table 2.

**Table 1:** Details of studies from which data were extracted for retrospective analyses

Study detail	NCT00601250 [18]	NCT00602472 [19]	NCT00621140 [20]	NCT00641043 [21]
Intervention	Once daily oral linagliptin 5 mg	Once daily oral linagliptin 5 mg	Once daily oral linagliptin 5 mg	Once daily oral linagliptin 5 mg
Co-medication	Metformin ≥ 1,500 mg/day	Metformin ≥ 1,500 mg/day + sulfonylurea at maximum tolerated dose	None	Once daily pioglitazone 30 mg
Objective	Efficacy and safety of linagliptin compared with placebo when added to metformin background therapy after 24 weeks of treatment	Efficacy and safety of linagliptin compared with placebo add-on therapy to metformin in combination with a sulfonylurea background therapy after 24 weeks of treatment	Efficacy and safety of linagliptin monotherapy compared with placebo after 24 weeks of treatment	Efficacy and safety of linagliptin in combination with pioglitazone 30 mg, compared with pioglitazone 30 mg monotherapy after 24 weeks of treatment

**Table 2:** Demographic and baseline characteristics

Characteristic	Placebo	Linagliptin			Total
		CC	CT	TT	
Number of patients	268	356	264	73	961
Sex, *n* (%)					
Male	132 (49.3)	170 (47.8)	121 (45.8)	45 (61.6)	468 (48.7)
Female	136 (50.7)	186 (52.2)	143 (54.2)	28 (38.4)	493 (51.3)
Race, *n* (%)					
White	185 (69.0)	205 (57.6)	205 (77.7)	59 (80.8)	654 (68.1)
Black	1 (0.4)	3 (0.8)	1 (0.4)	0 (0.0)	5 (0.5)
Asian	82 (30.6)	148 (41.6)	58 (22.0)	14 (19.2)	302 (31.4)
Mean age, years (SD)	56.7 (10.1)	57.5 (9.7)	57.3 (10.1)	58.7 (9.4)	57.3 (9.9)
Age groups, years, *n* (%)					
≤50	71 (26.5)	75 (21.1)	68 (25.8)	14 (19.2)	228 (23.7)
51 to <65	135 (50.4)	196 (55.1)	125 (47.3)	41 (56.2)	497 (51.7)
65 to <75	55 (20.5)	72 (20.2)	65 (24.6)	14 (19.2)	206 (21.4)
≥75	7 (2.6)	13 (3.7)	6 (2.3)	4 (5.5)	30 (3.1)
Mean baseline weight, kg (SD)	80.71 (16.74)	78.19 (17.82)	81.37 (16.19)	82.83 (17.64)	80.12 (17.12)
Mean baseline BMI, kg/m^2^ (SD)	29.74 (4.75)	29.09 (5.10)	30.12 (4.65)	29.12 (4.71)	29.56 (4.86)
Baseline BMI categorical, kg/m^2^, *n* (%)					
<25	47 (17.5)	84 (23.6)	40 (15.2)	14 (19.2)	185 (19.3)
25 to <30	103 (38.4)	132 (37.1)	95 (36.0)	30 (41.1)	360 (37.5)
≥30	118 (44.0)	140 (39.3)	129 (48.9)	29 (39.7)	416 (43.3)
Mean baseline HbA_1c_, % (SD)	8.2 (0.9)	8.2 (0.8)	8.2 (0.8)	8.1 (0.9)	8.2 (0.9)
Mean baseline HbA_1c_, mmol/mol (SD)	66.1 (9.8)	66.1 (8.7)	66.1 (8.7)	65.0 (9.8)	66.1 (9.8)
Baseline HbA_1c_, categorical %, *n* (%)					
<7.0	12 (4.5)	17 (4.8)	13 (4.9)	2 (2.7)	44 (4.6)
7.0 to <8.0	106 (39.6)	146 (41.0)	96 (36.4)	34 (46.6)	382 (39.8)
8.0 to <9.0	93 (34.7)	124 (34.8)	106 (40.2)	24 (32.9)	347 (36.1)
≥9.0	57 (21.3)	69 (19.4)	49 (18.6)	13 (17.8)	188 (19.6)

### Clinical objectives

The primary clinical endpoint in all four studies was change in HbA_1c_ (%) from baseline after 24 weeks of treatment, defined as difference between HbA_1c_ (%) at 24 weeks and HbA_1c_ (%) at baseline. A secondary objective in studies NCT00601250 [18] and NCT00621140 [20] was change from baseline in 2 h PPG.

### Genotype analysis

DNA was extracted from whole-blood samples and normalised to a standard concentration of 50 ng/μl. In addition, 92 blinded DNA samples from the same study were used to validate the detected genotypes. Locus-specific DNA fragments were amplified by PCR with 50 ng genomic DNA and 5 μmol/l each of forward and reverse primers. Purified PCR products were sequenced using the Sanger method [22] in a reaction containing 2 μmol/l sequencing primer and BigDye Terminator v3.1 (Life Technologies, Carlsbad, CA, USA). The sequencing primers were selected to detect the presence of the following *TCF7L2* variants in the sample: rs7903146 (C > T; intron), rs12255372 (G > T; intron), rs10885406 (A > G; intron) and rs731788 (C > G; near 3' region microRNA binding site).

### Statistical analyses

Data were pooled from all randomised patients from the four trials listed (Table 1) who were treated with at least one dose of study medication, had baseline measurements of HbA_1c_, had at least one measurement of HbA_1c_ while on treatment, had genetic polymorphism data available and passed the genetic mismatch quality control criteria. If the HbA_1c_ data after 24 weeks of treatment were not available, the last observed data point was carried forward for the analysis. The homogeneity of the treatment effect on the primary endpoint change in HbA_1c_ (%) from baseline after 24 weeks in the genotype subgroups was investigated using an ANCOVA. Baseline HbA_1c_ was the linear covariate, while washout period for prior oral glucose-lowering therapy (yes/no), treatment-genotype group (genotype groups CC, CT and TT for patients treated with linagliptin and the placebo group), race and study were set as fixed classification effects. Pairwise comparisons between homozygous non-risk (CC) and heterozygous risk (CT) or homozygous risk (TT) variant carriers receiving linagliptin as monotherapy or in combination with other anti-hyperglycaemic agents were also performed. To evaluate how representative the subgroup of patients with available pharmacogenomic data was with respect to their response to linagliptin therapy, the results obtained from these analyses were compared with those from the corresponding ANCOVA analyses on the pooled clinical data from all patients in the four studies. Demographic variables and baseline characteristics of the genotyped subpopulation were tested for differences between treatment–genotype groups (genotype groups CC, CT and TT for patients receiving linagliptin, and the placebo group) by *χ* ^2^ test, Fisher’s exact test or ANOVA.

Additional analyses were performed to address the potential influence of study, demographic and baseline characteristics by subgroup analyses as well as by incorporating them as additional factors or covariates into the ANCOVA model. Sensitivity analysis to investigate the impact of the last observation carried forward (LOCF) imputation was performed using only observed cases. SAS version 9.2 (SAS Institute, Cary, NC, USA) was used for all analyses.

## Results

### Demographic and baseline characteristics

Demographic variables and baseline characteristics were tested for differences between treatment–genotype groups (genotype groups CC, CT and TT for patients receiving linagliptin, and the placebo group). A significant difference was observed only for race (*p* < 0.0001) due to different frequencies of the *TCF7L2* risk allele in different ethnicities (see Table 3), so race was included into the ANCOVA model. The observed slight differences for baseline weight (*p* = 0.0443) and BMI (*p* = 0.0518) had no influence on the results when including them additionally into the ANCOVA model.

**Table 3:** Allelic and genomic frequencies of *TCF7L2* rs7903146 polymorphisms in the analysed population

Race	*n*	Minor allele frequency (%)	Genotype frequency (%)			Test for deviation from Hardy–Weinberg equilibrium (*p* value)
		T	CC	CT	TT	
Asian	302	18.5	68.9	25.2	6.0	0.0037
Black	5	20.0	60.0	40.0	0.0	0.5762
White	654	34.3	43.4	44.5	12.1	0.7371
All races	961	29.3	51.5	38.4	10.1	0.0235

### Distribution of allelic and genotype frequencies

The allelic and genotype frequencies of the various *TCF7L2* polymorphisms were determined for the cohort of consenting patients and the data categorised by race. The data for SNP rs7903146, which has the strongest association with type 2 diabetes, are shown in Table 3. The minor allelic frequency for the T allele was highest among white patients (34.3%) and lowest among Asian patients (18.5%), with black patients falling in the middle (20.0%). Homozygous TT occurred in 12.1% of white and 6.0% of Asian patients, but was absent in the black patients (Table 3), possibly owing to the low number of black patients participating in these studies. The observed frequencies in the white and Asian patients are concordant with publicly available allele and genotype frequencies [23, 24]. Results for the other polymorphisms and haplotypes were similar to those for rs7903146 (data not shown).

### Comparison of clinical response between genotyped subgroup and all patients

**Fig. 2:**
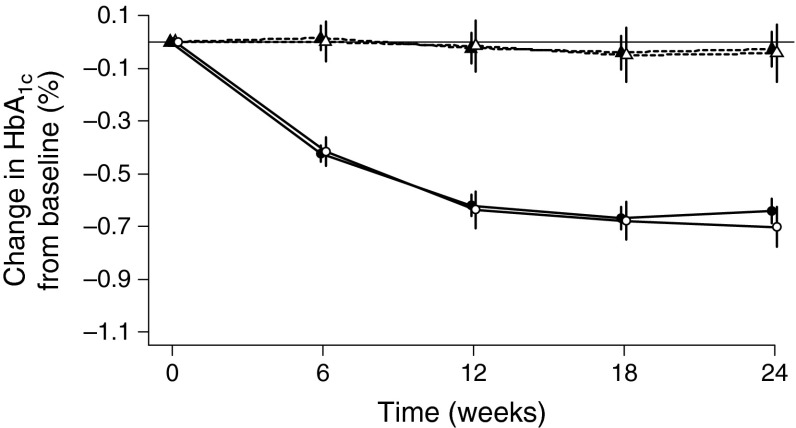
Adjusted mean difference between HbA_1c_ (%) at a given time and HbA_1c_ (%) at baseline (change in HbA_1c_ [%] from baseline) with 95% CIs for the whole cohort of patients from the four clinical trials and the subgroup for which pharmacogenomic data were available. ANCOVA model includes baseline HbA_1c_ as linear covariate and prior oral glucose-lowering therapy (yes/no), treatment, study and treatment-by-study interaction as fixed classification effects. Placebo complete clinical data, *n* = 728; placebo genotyped subgroup, *n* = 268; linagliptin complete clinical data, *n* = 1,876; linagliptin genotyped subgroup, *n* = 693. Black triangles, placebo complete clinical data; white triangles, placebo genotyped subgroup; black circles, linagliptin complete clinical data; white circles, linagliptin genotyped subgroup. To convert values for HbA_1c_ in DCCT % to mmol/mol, multiply by 10.929 and then subtract 23.50

To determine whether the subgroup for which genomic analysis was performed was representative of the population of patients who participated in all four trials, the clinical response observed for the subgroup was compared with that of the whole patient population (Fig. 2). The clinical responses to treatment with linagliptin or placebo in the subgroups for which pharmacogenomic data were available were essentially identical to those of the corresponding treatment groups in the whole cohort of patients from the four clinical trials—reduction in HbA_1c_ was similar in the two groups treated with linagliptin and HbA_1c_ did not change from baseline in the two groups receiving placebo. These results suggest that the pharmacogenomic subgroups were representative of the entire pooled patient population.

### Subgroup analyses

To address possible differences between the trials, we analysed each trial separately as well as in a pooled analysis again, incorporating additionally the study-by-treatment–genotype group interaction effect in the ANCOVA model. No significant heterogeneity of effects across the trials was observed. Detailed information and results of ANCOVA for each specific trial in comparison to the whole study population are given in electronic supplementary material (ESM) Table 1.

### Association of genotype with clinical response

**Fig. 3:**
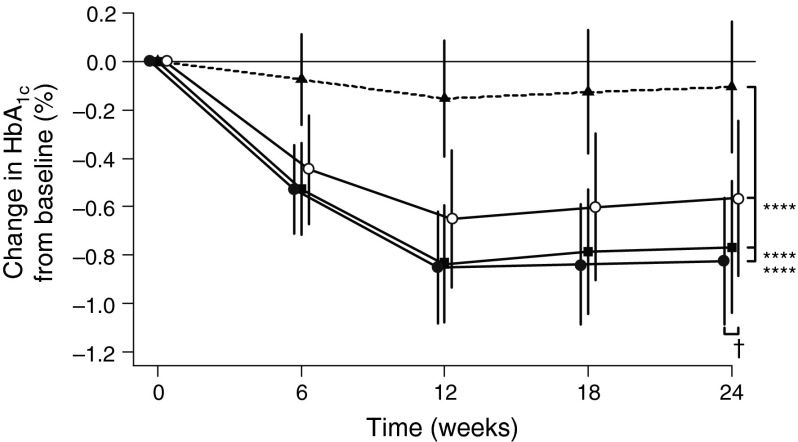
*TCF7L2* rs7903146 genotype-associated adjusted mean difference between HbA_1c_ (%) at a given time and HbA_1c_ (%) at baseline (change in HbA_1c_ [%] from baseline) with 95% CIs. Linagliptin CC, *n* = 356; linagliptin CT, *n* = 264; linagliptin TT, *n* = 73; placebo, *n* = 268. ****p < 0.0001 vs placebo at 24 weeks; ^†^ *p* < 0.05, linagliptin-treated homozygous CC vs homozygous TT patients at 24 weeks. Black circles, linagliptin CC; black squares, linagliptin CT; white circles, linagliptin TT; black triangles, placebo. To convert values for HbA_1c_ in DCCT % to mmol/mol, multiply by 10.929 and then subtract 23.50

Based on *TCF7L2* rs7903146 genotype, patients receiving linagliptin therapy were categorised into three groups: CC (homozygous non-risk allele carrier), CT (heterozygous) and TT (homozygous risk allele carrier). The strongest, almost identical, response to linagliptin therapy (i.e. lowering of HbA_1c_) was observed in patients with homozygous CC (*n* = 356; *p* < 0.0001) and heterozygous CT (*n* = 264; *p* < 0.0001) genotypes with a decrease of 0.82% (9.0 mmol/mol) and 0.77% (8.4 mmol/mol) in HbA_1c_, respectively, compared with baseline (Fig. 3). A less robust, but still statistically (*p* = 0.0006) and clinically significant, decrease of 0.57% (6.2 mmol/mol) in HbA_1c_ in response to linagliptin therapy after 24 weeks was observed in patients who were homozygous TT (*n* = 73). The difference in response to linagliptin therapy between homozygous TT patients and homozygous CC patients was statistically significant (*p* = 0.0182).

The comparison of the number of patients in LOCF analyses vs the number of patients with observed 24 week data indicate that we have >80% observed cases in the linagliptin-treated group and >70% in the placebo group. The results, based only on observed cases, showed a significant difference of ~0.23% (*p* = 0.0392) between linagliptin-treated homozygous TT and non-risk-carrier CC patients and this is in concordance with the results obtained in the analyses for the LOCF set.

### Association of genotype with PPG levels

Patients receiving placebo showed an increase from baseline in 2 h PPG levels with a mean rise of 2.13 mmol/l (ESM Fig. 1), which is likely related to the washout of previous glucose-lowering agents in the two clinical studies NCT00601250 [18] and NCT00621140 [20]. This was in accordance with an increase in HbA_1c_ levels in the placebo group in those two studies. Similar to the observed HbA_1c_ levels after treatment, all patients treated with linagliptin showed a decrease from baseline in 2 h PPG levels after 24 weeks. Patients homozygous for the wild-type allele (CC) showed the greatest decrease from baseline in 2 h PPG levels, with a mean decrease of 2.78 mmol/l. Although there was greater variability in patients heterozygous for the risk allele (CT), the reduction from baseline in 2 h PPG levels was similar to that observed in the homozygous CC patients, with a mean decrease of 2.55 mmol/l. Patients homozygous for the risk allele (TT) showed the smallest decrease from baseline in 2 h PPG levels (1.65 mmol/l), although it should be noted that only a few patients’ data (*n* = 6) was available for this group.

## Discussion

The present studies have been undertaken to assess the impact of *TCF7L2* genotypes on the response to incretin-based therapy, for the first time in a longitudinal setting. This is important because genetic polymorphism has been suggested to contribute to the susceptibility of individuals to environmental stimuli, resulting in increased prevalence of diabetes. Of the many genes investigated, *TCF7L2*, a β-catenin bipartite transcription factor, integral to the upregulation of incretin secretion from intestinal endocrine L cells and the proliferation of pancreatic beta cells [25–27], has the strongest known association with diabetes [5, 6]. The high-risk genotypes of *TCF7L2* SNPs rs7903146 and rs12255372 are strongly associated with reduced insulin secretion, possibly owing to impaired response to incretins [10, 11] and impaired beta cell function [10, 12, 13]. Accordingly, we tested the hypothesis that the efficacy response to linagliptin therapy, which acts via inhibition of incretin degradation, may be reduced in patients with type 2 diabetes who have high-risk *TCF7L2* genotypes. It is possible that these individuals may be genetically predisposed to produce and secrete less incretin or have an impaired incretin response compared with those with wild-type genotype.

As expected, in the pooled analyses, the HbA_1c_ levels of patients showed no change from baseline when administered placebo. In response to treatment with linagliptin, wild-type homozygous patients exhibited a robust −0.82% (−9.0 mmol/mol) reduction from baseline in HbA_1c_ levels (*p* < 0.0001). In contrast, the response to treatment with linagliptin in patients who were homozygous for the risk allele was reduced (−0.57% [−6.2 mmol/mol] decrease from baseline in HbA_1c_ on average; *p* < 0.0006), but still clinically meaningful (>0.5% [>5.5 mmol/mol] decrease).

Similar to the observations for HbA_1c_, homozygous wild-type patients treated with linagliptin showed a decrease in 2 h PPG levels compared with patients receiving placebo. Heterozygous patients exhibited a response similar to that observed for homozygous wild-type patients and homozygous risk carriers (TT) showed the least decrease from baseline in 2 h PPG levels. However, the number of patients in each of these groups was small and probably not sufficient to allow meaningful conclusions to be made.

The observed differences in linagliptin efficacy response of ~25% between *TCF7L2* homozygous risk carriers (12% of whites) and non-risk carriers are in line with previous data of an association of *TCF7L2* and sulfonylurea response [9]. This would support the recent postulation by Schäfer et al [28] that *TCF7L2* variants are associated with a functional defect in the beta cells. Considering that the efficacy response to linagliptin in the TT carriers was clinically relevant, it is intriguing to speculate that a stronger loss of efficacy than that observed in this investigation would have been expected in homozygous carriers if a specific incretin-related defect was present. Based on present data, this cannot be ruled out. Another possibility for the lack of a more pronounced effect may be that polymorphisms in a single gene may not be sufficient to produce a significant change in a patient’s response to a DPP-4 inhibitor. Variants in additional genes could potentially contribute to inter-individual variability in response, and combined analyses of several risk genes for type 2 diabetes implicated in the regulation of beta cell function may further help explain the variability of efficacy response to insulin secretagogues.

The study has some limitations, mainly related to the relatively small sample size. This was addressed by combining the data from four clinical trials. However, we cannot completely rule out the influence of co-medication. The fact that we did observe the same trend for the differences in response to linagliptin treatment between CC wild-type and TT risk carriers by analysing each trial with different background therapies separately, supports our hypothesis. In addition, a disease–genetic process in *TCF7L2* carriers could contribute to the effect, but the change in HbA_1c_ level from baseline in the placebo groups did not reveal differences between CC and TT risk allele carriers, indicating a pharmacogenetic effect.

Nevertheless, results must be interpreted with some caution and should ideally be confirmed in a second cohort. Since linagliptin was the only DPP-4 inhibitor evaluated, it is unknown whether or not these observations are specific to linagliptin or whether they can be regarded as a class effect.

In conclusion, to our knowledge, these are the first studies testing the impact of *TCF7L2* genotype on the response to incretin therapy (DPP-4 inhibitor) in a longitudinal cohort. Our analyses demonstrate for the first time that although the clinical response to the DPP-4 inhibitor linagliptin was somewhat attenuated in homozygous *TCF7L2* risk carriers, this treatment results in a clinically meaningful glucose-lowering potency, even in homozygous high-risk allele carrier patients.

## Electronic supplementary material

Below is the link to the electronic supplementary material.

ESM Table 1 (PDF 38 kb)

ESM Fig. 1 (PDF 292 kb)

## Abbreviations

DPP-4: Dipeptidylpeptidase-4
PPG: Postprandial plasma glucose
SNP: Single-nucleotide polymorphism
