# Supplementary material for: Influence of TCF7L2 gene variants on the therapeutic response to the dipeptidylpeptidase-4 inhibitor linagliptin
Source: Diabetologia. 2014 Jun 7;57(9):1869–75. doi: 10.1007/s00125-014-3276-y (PMC4119242; doi:10.1007/s00125-014-3276-y)
Supplement: Supplementary file 1 — (PDF 38 kb) [file 125_2014_3276_MOESM1_ESM.pdf]

ESM Table 1 Detailed information and results of each specific trial in comparison to the whole study population

	NCT00641043	NCT00621140	NCT00601250	NCT00602472	Pooled
Brief description	Initial combination with pioglitazone	Efficacy of monotherapy vs. placebo at 24 weeks	Efficacy as add-on to metformin vs. placebo	Efficacy as add-on to metformin/SU vs placebo	
Randomization	1:2	1:2	1:3	1:3	
Total number of patients in the study, *N* (%)	380 (100.00)	496 (100.00)	688 (100.00)	1040 (100.00)	2604 (100.00)
Placebo	128 (33.68)	163 (32.86)	175 (25.44)	262 (25.19)	728 (28.96)
Treated	252 (66.32)	333 (67.14)	513 (74.56)	778 (74.81)	1876 (72.04)
Total number of genotyped patients, *n* (%)	133 (35.00)	179 (36.09)	210 (30.52)	439 (42.21)	961 (36.90)
Placebo	50 (39.06)	62 (38.04)	54 (30.86)	102 (38.93)	268 (36.81)
Treated	83 (32.94)	117 (35.14)	156 (30.41)	337 (43.32)	693 (36.94)
Genotype distribution in treated patients, *n* (%)					
CC	34 (40.96)	51 (43.59)	78 (50.00)	193 (57.27)	356 (51.37)
CT	40 (48.19)	54 (46.15)	64 (41.03)	106 (31.45)	264 (38.10)
TT	9 (10.84)	12 (10.26)	14 (8.97)	38 (11.28)	73 (10.53)
Frequency of minor allele T, %	34.94	33.33	29.49	27.00	29.58

Baseline HbA_{1c}, mean % (SD)					
Placebo	8.70 (0.96)	8.05 (0.97)	7.99 (0.91)	8.23 (0.81)	8.23 (0.93)
Treated by genotype					
CC	8.48 (0.76)	7.88 (0.74)	8.03 (0.94)	8.22 (0.81)	8.15 (0.84)
CT	8.41 (0.71)	7.89 (0.86)	8.30 (0.89)	8.17 (0.79)	8.18 (0.83)
TT	8.52 (0.53)	7.75 (1.27)	8.16 (0.94)	8.09 (0.85)	8.10 (0.92)
Results of ANCOVA[1]					
Adjusted change in HbA_{1c} from baseline, mean % (95% CI)					
Placebo	–0.37 (–0.95, 0.21)	0.15 (–0.06, 0.36)	0.34 (–0.11, 0.78)	–0.24 (–0.63, 0.16)	–0.11 (–0.38, 0.16)
Treated by genotype					
CC	–0.91 (–1.52, –0.30)	–0.62 (–0.85, –0.39)	–0.58 (–0.97, –0.20)	–0.94 (–1.33, –0.55)	–0.82 (–1.09, –0.56)
CT	–0.92 (–1.50, –0.34)	–0.56 (–0.79, –0.33)	–0.50 (–0.94, –0.07)	–0.88 (–1.28, –0.48)	–0.77 (–1.04, –0.50)
TT	–0.91 (–1.66, –0.16)	–0.12 (–0.59, 0.36)	–0.30 (–0.91, 0.32)	–0.70 (–1.16, –0.25)	–0.57 (–0.89, –0.24)
Difference in change in HbA_{1c} from baseline: treated CC vs. TT, adjusted mean % (95% CI); *P* value	–0.00 (–0.57,0.57)<br>0.9915	–0.51 (–1.03,0.02)<br>0.0577	–0.28 (–0.82, 0.25)<br>0.2984	–0.24 (–0.53, 0.05)<br>0.1041	–0.26 (–0.48, –0.04)<br>0.0182

[1]NCT00641043, NCT00621140, NCT00601250: ANCOVA model includes continuous baseline HbA_{1c}, number of prior oral glucose-lowering drugs (OAD), race, treatment-genotype group; NCT00602472: ANCOVA model includes continuous baseline HbA_{1c}, race, treatment-genotype group; pooled analysis of all studies: ANCOVA model includes continuous baseline HbA_{1c}, wash-out-period for prior OADs (yes/no), race, study, treatment-genotype group.
