# Supplementary material for: Influence of TCF7L2 gene variants on the therapeutic response to the dipeptidylpeptidase-4 inhibitor linagliptin
Source: Diabetologia. 2014 Jun 7;57(9):1869–75. doi: 10.1007/s00125-014-3276-y (PMC4119242; doi:10.1007/s00125-014-3276-y)

ESM Fig. 1 Box plot for change from baseline of 2h-PPG at week 24. Whiskers indicate sample minimum and maximum and dots represent mean values

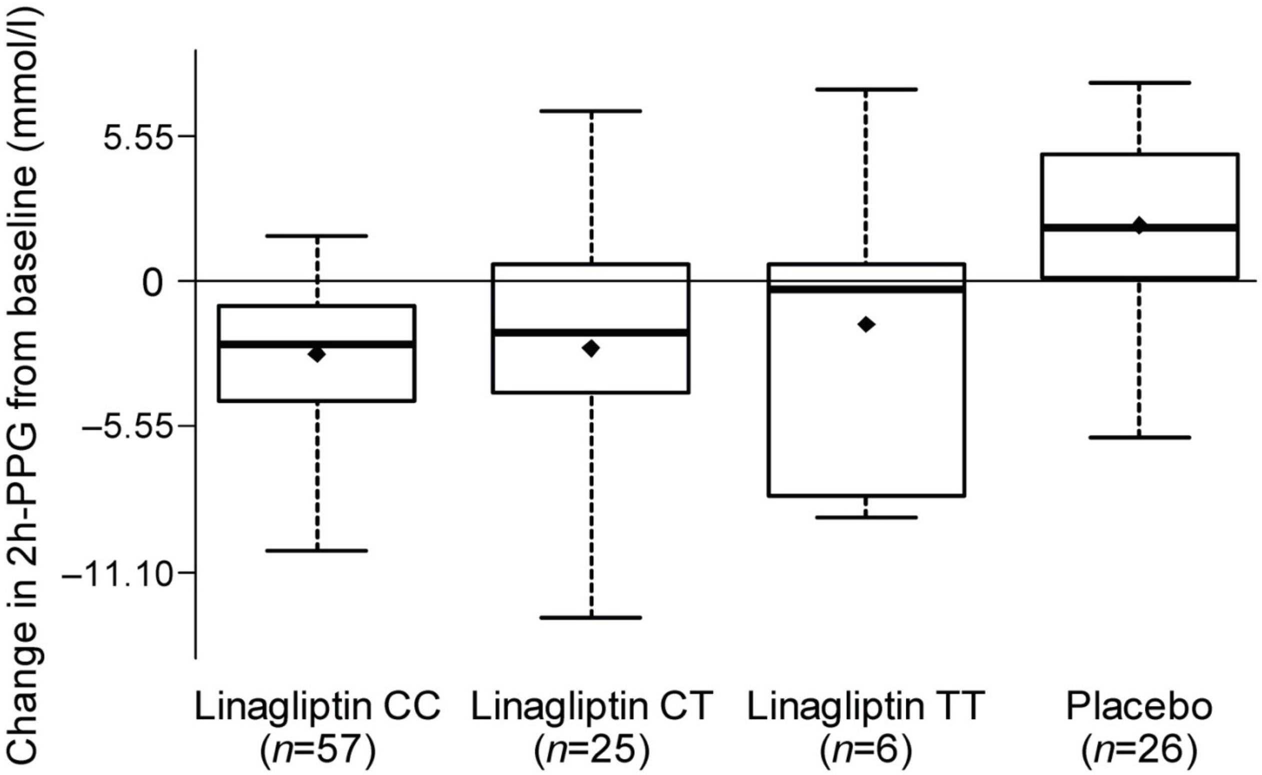

Supplement: Supplementary file 2 — (PDF 292 kb) [file 125_2014_3276_MOESM2_ESM.pdf]
